# Evaluation of In Vitro Cytotoxic, Genotoxic, Apoptotic, and Cell Cycle Arrest Potential of Iron–Nickel Alloy Nanoparticles

**DOI:** 10.3390/toxics10090492

**Published:** 2022-08-24

**Authors:** Özgür Vatan

**Affiliations:** Department of Biology, Faculty of Arts and Science, Görükle Campus, Bursa Uludağ University, 16059 Nilüfer, Bursa, Turkey; ovatan@uludag.edu.tr

**Keywords:** iron-nickel alloy nanoparticles, cytotoxicity, genotoxicity, apoptosis, cell cycle arrest, ROS

## Abstract

The use of iron-nickel alloy nanoparticles (Fe-Ni ANPs) is increasing daily in various fields. People are increasingly exposed to these nanoparticles for occupational and environmental reasons. Our study determined some of the effects of Fe-Ni ANP exposure and impacts on human health at the cellular level. The cytotoxic and genotoxic potentials of Fe-Ni ANPs were investigated by XTT, clonogenic, comet, and GammaH2AX analyses using Beas-2B cells. Annexin V, multicaspase, and cell cycle arrest methods were used to understand the apoptotic mechanism of action. The intracellular ROS method was used to determine the primary mechanism that leads to cytotoxic and genotoxic activity. The Fe-Ni ANPs showed cytotoxic activity with the XTT and clonogenic methods: they had genotoxic potential, as demonstrated via genotoxicity methods. It was determined that the cytotoxic effect was realized by the caspase-dependent apoptotic pathway, and the cells were stopped at the G0/G1 stage by Fe-Ni ANPs. Increased intracellular ROS due to Fe-Ni ANPs led to cytotoxic, genotoxic, and apoptotic activity. Potential risks to human health due to Fe-Ni ANPs were then demonstrated at the cellular level.

## 1. Introduction

Nanotechnology is one of the most promising technologies of the 21st century. Magnetic nanoparticles such as Fe-Ni (iron-nickel) are attractive because of their different uses [1]. Many studies have shown that magnetic nanoparticles have a wide range of uses. These examples include Khan et al. [2] with magnetic fluid recording, catalysis, biotechnology/biomedicine, materials sciences, photocatalysis, electrochemical and bioelectrochemical sensing, microwave absorption, magnetic resonance imaging, medical diagnosis, data storage, and environmental remediation. Fe-Ni can also be an electrode for supercapacitors and lithium-ion batteries. Sun et al. [3] emphasized that magnetic nanoparticles could revolutionize clinical diagnostic and therapeutic techniques. Due to the properties of magnetic nanoparticles, their potential as magnetic resonance imaging contrast agents and as carriers for targeted drug delivery has attracted attention [3,4,5,6]. Iron alloy nanoparticles have potential applications in different research areas due to their properties in electromagnetic shielding, magnetism, and catalysis [7,8,9,10,11,12].

It has also been stated that Fe-Ni nanoparticles are attractive candidates for magnetic cores of transformers and inductors operated at high frequencies [13]. Recently, it has been demonstrated that hydrogen obtained from water electrolysis can be used to store energy from renewable energy sources, such as the sun and wind. Studies supporting the use of Fe-Ni nanoparticles as catalysts during the oxygen evolution reaction in water electrolysis are increasing [14,15,16,17]. Fe-Ni nanoparticles can also remove pollutants, such as Cr(VI) [18], triclosan [19], trichloroethane [20], malachite green [21], dichlorophenol [22], nitrates [23], and tetracycline [24], from wastewater or potable water. Hassanein et al. [25] showed that the addition of nanoparticles, such as Fe, Ni, or Co, to anaerobic digestion reactors where organic wastes are used increases the formation of CH_4_, which can be used as a renewable energy source.

Nanotoxicology studies are increasing rapidly due to concerns about human health and environmental effects of nanoparticle exposure during production and subsequent use. Souza et al. [26] investigated the cytotoxic and genotoxic activity of samples containing Fe and Ni and many other elements consisting of particles smaller than 10 microns in MRC-5 cells in samples collected from areas with intense air pollution. They showed that the nanoparticles in their study increased the cytotoxic activity. Lu et al. [27] investigated the cytotoxic activity of samples collected from polluted air areas containing Fe, Ni, and other elements in A549 cells with the MTT (3-(4,5-dimethylthiazol-2-yl)-2,5-diphenyltetrazolium bromide) test and found similar results. Machado et al. [28] investigated the cytotoxic effect of ballistic aerosol, which contains W-Fe-Ni and W-Fe-Co ANPs, on A549 cells. Bhushan et al. [29] evaluated the cytotoxic activity of α-Fe_2_O_3_/NiO nanocomposites by the MTT method in MCF10a and MCF7 cells. The cytotoxic activity increased in both studies. Jiang et al. [30] stated that Fe-Ni and Fe nanoparticles reduced cell growth and denitrification rates in *Paracoccus* sp Strain YF1.

The industrial use of iron-nickel alloy nanoparticles (Fe-Ni ANPs) causes occupational exposure in this industry. Industrial use can lead to environmental pollution. Studies investigating the nanotoxic and nanogenotoxic activities of Fe-Ni ANPs seem limited despite their wide use. Thus, we investigated the cytotoxic, genotoxic, apoptotic, cell cycle arrest, and generation of intracellular reactive oxygen species (ROS) via Fe-Ni ANPs in Beas-2B cells (human lung bronchial epithelial cells).

## 2. Materials and Methods

### 2.1. Chemical and Reagents

XTT (2,3-bis(2-methoxy-4-nitro-5-sulfophenyl)-5-carboxanilide-2H-tetrazolium)-based cell proliferation kit, Fetal Bovine Serum, Penicillin Streptomycin solution, and Trypsin-Edta solution were purchased from Biological Industries (Kibbutz Beit Haemek, Israel). Muse^®^ Annexin V & Dead Cell Kit, Muse^®^ Cell Cycle Kit, Muse^®^ MultiCaspase Kit, and Muse^®^ H2A.X Activation Dual Detection Kit were purchased from Luminex (Austin, TX, USA). Fe-Ni ANPs (55% Fe, 45% Ni) and all other chemicals and reagents were purchased from Sigma-Aldrich (Darmstadt, Germany).

### 2.2. Characterization of Fe-Ni ANPs

Before each application, stock solutions were freshly prepared from Fe-Ni ANPs in distilled water with a 1 mg/mL final concentration. This master stock solution was ultrasonicated (Bandelin Electronic, Sonifier, Berlin, Germany) for 20 min before each application, and application concentrations were obtained by making the necessary dilutions. Particle, size, morphology, and agglomeration states were characterized using transmission electron microscopy (FEI, Tecnai Spirit G2, 120 kV, Hillsboro, OR, USA) (TEM). The size of 200 random particles in the micrographs was determined using an image analysis system (Arganit Mikrosistem, Istanbul, Turkey) via the measurement scale. Intracellular images of Fe-Ni ANPs were also obtained by transmission electron microscopy (Hitachi HT7800, Tokyo, Japan). Average hydrodynamic size, size distribution, and zeta potential of particles in suspension were determined by dynamic light scattering (DLS) using a Nano-ZS90 Malvern Zetasizer. Fe-Ni ANPs crystallographic structures were determined by X-ray diffraction (XRD) (Malvern Panalytical EMPYREAN, Malvern, UK). Further, d-spacing values and Miller indices were calculated by the device’s software based on Bragg’s Law [31]. The average crystallite size was measured by the Scherrer equation (λ = 0.154) [32]. 

### 2.3. Cell Culture

Beas-2B was provided by ATCC, CRL-9609 cells, which are healthy human bronchial epithelial cells, and used in our study as it was emphasized in previous studies that it is suitable for investigating the toxicity of various chemicals, including nanoparticles, and inhalation is known to be an essential form of exposure for multiple nanoparticles [33,34,35]. The cells were grown in RPMI-1640 medium supplemented with 10% fetal bovine serum (FBS), penicillin-streptomycin (50 µg/mL), 2 mM L-glutamine, and 1% sodium pyruvate. Cells were maintained at 37 °C in a humidified atmosphere containing 5% CO_2_, grown in 75 cm^2^ flasks, and subcultured once a week.

### 2.4. Cytotoxicity

#### 2.4.1. XTT Assay

XTT, a tetrazolium salt, is reduced by metabolically active cells to an orange formazan dye [36]. XTT-based cell proliferation kit was used to determine the cytotoxic effect of Fe-Ni ANPs. The method was applied taking into account the kit manufacturer’s instructions and the points outlined by Stone et al. [37]. Briefly, after standard trypsinization, the cells were seeded at 5 × 10^3^ cell/well in 96-well plates for XTT tests. After 24 h of culture in normal growth medium, the cells were exposed to different concentrations of Fe-Ni ANPs (0.1; 0.5; 1; 2; 4; 8; 16; 32; 64; 128 µg/mL) for 24 h. The positive control was treated with 3 mM H_2_O_2_. After treatment, the wells were washed with PBS, and the medium was replaced with a fresh medium (100 µL). The activated XTT solution (50 µL) was added to each well according to the manufacturer’s instructions. The plate was incubated for an additional 4 h in the CO_2_ incubator at 37 °C. The absorbance was determined using a microplate reader at a wavelength of 450 nm. All of the analyses were performed in triplicate. After blank subtraction, the results were expressed as a percentage of the untreated control [37]. The absorbance of the untreated control cells was set to 100%, and the percent growth inhibition of cells was calculated as follows [38]:

(1 − A_exp_ group/A_control_) × 100

XTT results were shown as mean values of cell viability % ± standard deviation (SD). The half-maximal inhibitory concentration (IC50) value was calculated from the equation of function of the cell viability % curve consisting of at least three points. The IC50 was given as a mean value ± SD.

#### 2.4.2. Clonogenic Assay

The clonogenic assay is also known as the colony formation assay. Although it is used to determine the cell reproductive death effect of ionizing radiation, which is based on the ability of a single cell to form a colony, it is also used to determine the effectiveness of other cytotoxic agents [39]. Fifty thousand cells were seeded in T25 flasks and allowed to grow for 48 h. After this period, the cells were exposed to different concentrations of Fe-Ni ANPs (0.1; 0.5; 1; 2; 4; 8; 16; 32; 64; 128 µg/mL) for 24 h. The positive control was treated with 3 mM H_2_O_2_. After standard trypsinization, live cells were counted with trypan blue solution by Cedex XS Cell Analyzer (Roche Innovatis, Bielefeld, Germany). From each concentration group, 4 Petri dishes (60 × 15 mm) were seeded with 500 live cells/Petri. Cells were incubated for 12 days so that they could form colonies. Colonies formed at the end of the period were fixed with 100% methanol and stained with crystal violet. Colonies of at least 50 cells were counted in each Petri dish. The mean number of colonies in the untreated control group (L) and each concentration group (N) in the 4 Petri dishes prepared were determined, and the percent growth of cells was calculated as follows:

(N/L) × 100

All of the analyses were performed in triplicate. Clonogenic assays results were shown as mean values of cell viability % ± standard deviation (SD). The IC50 value was calculated from the equation of function of the cell viability % curve consisting of at least three points, and the IC50 was given as a mean value ± SD.

### 2.5. Genotoxicity 

#### 2.5.1. Comet Assay

Alkaline comet assay was performed according to Singh et al. [40] with some modifications [41] for mainly single-strand DNA breaks. Cells were seeded on T25 flasks at a density of 5 × 10^4^ cells/flask and allowed to grow for 48 h. After incubation, cells were exposed to different concentrations of Fe-Ni ANPs (1/4 IC50 represents IC12.5; ½ IC50 represents IC25; IC50; 3/2 IC50 represents IC75, based on an average of the IC50 values of the XTT and clonogenic assay) for 24 h. H_2_O_2_ (200 µM) was used as a positive control. After treatments, cells were suspended in 0.8% low melting agarose, prepared with PBS, spread on microscope slides previously coated with normal melting agarose, and allowed to solidify for 15 min at 4 °C. Slides were placed in a freshly prepared ice-cold lysis solution (2.5 M NaCl, 100 mM Na_2_EDTA, 10 mM Tris (pH 10), with 1% Triton X-100 (added just before use)) to lyse the cells overnight in the dark at 4 °C. Slides were subjected to DNA unwinding for 20 min, and then electrophoresis was performed at 0.7 V/cm and 300 mA for 25 min in freshly prepared cold electrophoresis buffer (1 mM EDTA sodium salt and 300 mM NaOH, pH 13). After electrophoresis, the slides were immersed in neutralization buffer (0.5 M Tris-HCl, pH 7.5) for 10 min. Finally, the slides were stained with 100 mL of ethidium bromide (20 mg/mL). Two slides were prepared for each sample, and 100 comets visual were scored from each slide. The slides were scored at a final magnification of 200 X using an image analysis system attached to a microscope (Nikon Eclipse 80i) equipped with a fluorescence attachment of a charge-coupled device (CCD) camera (Kameram 21). The extent of DNA damage was expressed as a measure of the tail length, percentage of DNA in the comet tail, and Olive tail moment. All analyses were performed in triplicate, and results were shown as mean values of comet parameters ± standard deviation (SD).

#### 2.5.2. Gamma-H2AX Assay

H2AX is a member of the histone H2A family. Specific phosphorylation of H2AX (γH2AX) is an important indicator of DNA damage, especially double-strand breaks [42]. The assay was used to detect double-stranded DNA breaks. According to the manufacturer’s instructions, the assay was performed using Muse^®^ H2A.X Activation Dual Detection Kit and Guava^®^ Muse^®^ Cell Analyzer (Luminex, Austin, TX, USA). Briefly, cells were seeded on T25 flasks at a density of 5 × 10^4^ cells/flask and allowed to grow for 48 h. After incubation, cells were exposed to different concentrations of Fe-Ni ANPs (IC25; IC75 as described in the comet assay) for 24 h. After trypsinization, cells were placed in fixation buffer and then permeabilized. Cell suspensions (including 200,000 cells) were mixed with antibody cocktails and incubated for 30 min at room temperature (dark). Cells were resuspended in assay buffer and analyzed on a Muse Cell Analyzer. All analyses were performed in triplicate, and results were shown as mean values of γH2AX kit parameters ± standard deviation (SD).

### 2.6. Cell Death Mode

#### 2.6.1. Annexin V Analysis

Annexin V can be measured with fluorescence markers and binds with high affinity to phosphatidyl serine translocated outside the cell membrane in early apoptotic cells. The increased membrane permeability in late apoptotic cells causes fluorescent 7-aminoactinomycin-D (7-AAD) to bind to GC regions in DNA [43,44]. Thus, we determined the mechanism of cell death using Muse^®^ Annexin V & Dead Cell Kit (Luminex Austin, TX, USA) and the Muse^®^ Cell Analyzer (Luminex Austin, TX, USA). Cells were seeded on T25 flasks at a density of 5 × 10^4^ cells/flask and allowed to grow for 48 h. After incubation, cells were exposed to different concentrations of Fe-Ni ANPs (IC25; IC75 as described in the comet assay) for 24 h. The assay was then performed according to the manufacturer’s instructions. All analyses were performed in triplicate, and results were shown as mean values of Annexin V kit parameters ± standard deviation (SD).

#### 2.6.2. Multicaspase Analysis

Caspases (cysteinyl-directed aspartate-specific proteases) play an essential role in propagating apoptotic signals in cells by activation [45,46]. The Muse^®^ MultiCaspase Kit (Luminex Austin, TX, USA) and Muse^®^ Cell Analyzer (Luminex Austin, TX, USA) can detect activated caspases (caspase-1, 3, 4, 5, 7, 8, and 9), and we determined whether cell death was mediated by the caspase-dependent apoptotic pathway. The assay was performed according to the manufacturer’s instructions after the preliminary steps described in the Annexin V assay above. All analyses were performed in triplicate, and the results are shown as mean values of multicaspase kit parameters ± standard deviation (SD).

### 2.7. Cell Cycle Arrested Analysis

The cell cycle arrest effect of Fe-Ni ANPs was evaluated using Muse^®^ Cell Cycle Kit and Muse^®^ Cell Analyzer. The assay was performed according to the manufacturer’s instructions after the preliminary steps described in the Annexin V assay above. All analyses were performed in triplicate, and the results are shown as mean values of cell cycle kit parameters ± standard deviation (SD).

### 2.8. Measurement of Intracellular ROS

Intracellular ROS level was determined using 2′, 7′-dichlorodihydrofluorescein diacetate (DCFH-DA) that can diffuse into the cell. DCFH-DA is deacetylated by cellular esterase and converted to non-fluorescent 2′, 7′-dichlorodihydrofluorescein. It is then oxidized to fluorescent 2′, 7′-dichlorodihydrofluorescein (DCF) by intracellular ROS. The measured fluorescence intensity is proportional to the level of ROS in the cell [47]. After standard trypsinization, the cells were seeded at 8 × 10^3^ cells/well in 96-well black plates for 24 h. The cells were washed with PBS thrice after 24 h of culture in a normal growth medium. After the wash, cells were incubated at 50 µM DCFH-DA with a serum-free medium for 4 h. Excess DCFH-DA was removed by washing three times with PBS, and DCFH-DA-loaded cells were obtained. The cells were exposed to different concentrations of Fe-Ni ANPs (0.1; 0.5; 1; 2; 4; 8; 16; 32; 64; 128 µg/mL) for 24 h. Fluorescence values were determined kinetically at 37 °C with a fluorometric plate reader (Fluoroskan Ascent FL 2.6, Thermo Fisher Scientific, Waltham, MA, USA) at 480 nm/530 nm. The experiment was performed in triplicate. The results are given as the mean relative fluorescence unit (RFU) ± SD 6, 12, 18, and 24 h (after subtracting background values caused by Fe-Ni ANPs in the absence of dye).

### 2.9. Statistical Analyses

Statistical analyses were performed using SPSS 22 software. After assessing the normality of the data distribution, using the one-sample Kolmogorov-Smirnov test, both parametric and nonparametric tests were used to detect differences at the 0.05 level of significance. Comet assay data were analyzed using Mann-Whitney U test. Other data were analyzed using one-way ANOVA (analyses of variance) with Tukey HSD (honestly significant difference) or Tamhane’s T2 post hoc test.

## 3. Results

### 3.1. Characterization of Nanoparticles

**Figure 1:**
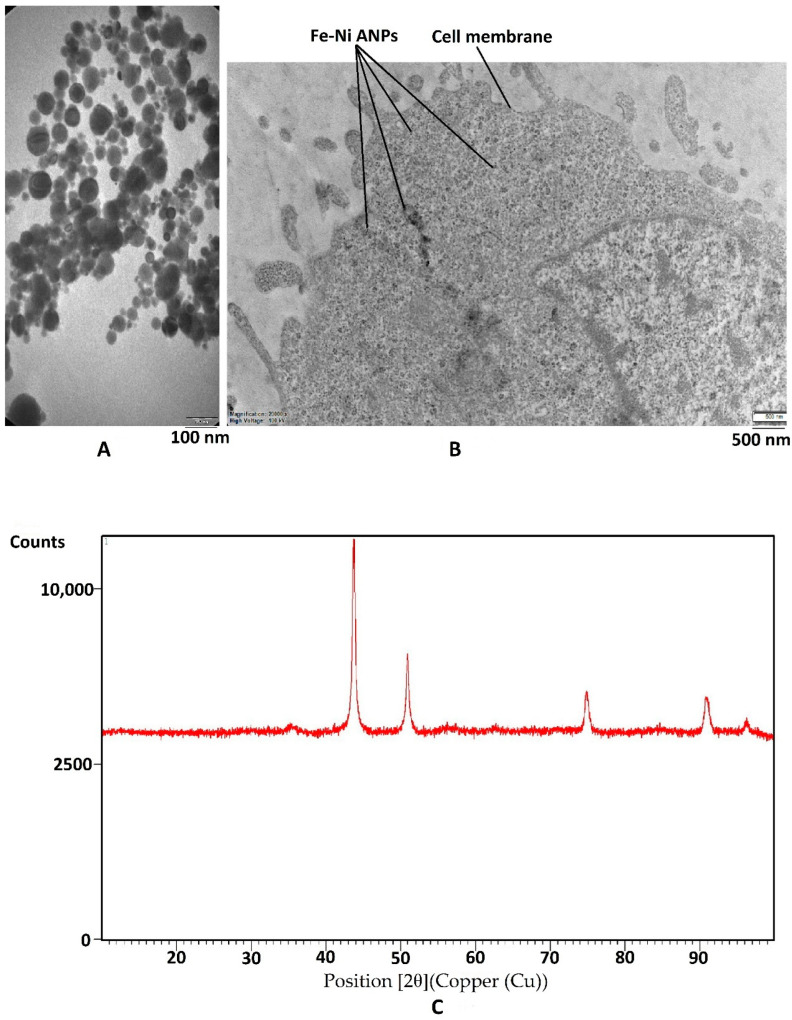
Characterization of nanoparticles. (**A**) Sample TEM image of Fe–Ni ANPs. (**B**) Sample intracellular TEM image of Fe-Ni ANPs. (**C**) XRD pattern of Fe-Ni ANPs.

The Fe-Ni ANPs zeta potential value of the suspension was determined as −24.8 mV. This value changed the −20.2 mV in the RPMI-1640 medium. Dynamic light scattering (DLS) analyses showed that the particle size was approximately 180 nm. TEM micrograph (Figure 1A) measurements were established to show that the particle size mean was 42.255 ± 17.708 nm (min = 11.896 nm; max = 101.515 nm). In the intracellular TEM analysis, it was observed that Fe-Ni ANPs entered the cell and were concentrated in the cytoplasm (Figure 1B). As a result of XRD analysis, no peaks belonging to the Fe or Ni phase were observed. The 2θ values, d-spacing, and Miller indices of the observed peaks are, respectively: 43.73, 2.1 Å (111); 50.932, 1.8 Å (200); 74.889, 1.3Å (220); 90.926, 1.1 Å (311) (Figure 1C). The average crystallite size was measured to be 17.679 nm by XRD.

### 3.2. Cytotoxicity

**Figure 2:**
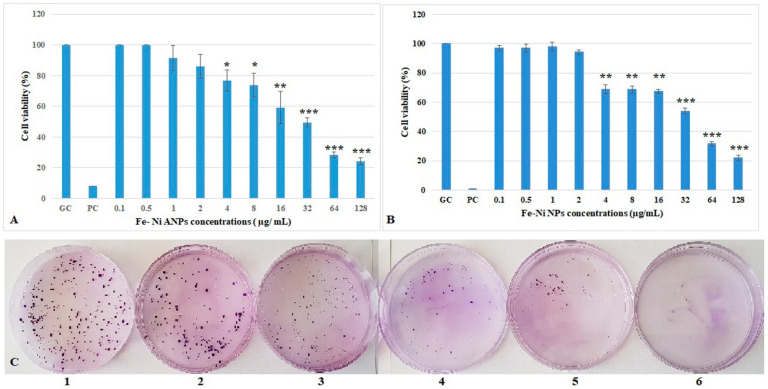
Viability of Beas-2B cells treated with Fe-Ni ANPs for 24 h. (**A**) Results from XTT. (**B**) Results from clonogenic assay. (**C**) Sample images of the clonogenic assay. **1**: growth control; **2**: 1 µg/mL Fe-Ni ANPs; **3**: 2 µg/mL Fe-Ni ANPs; **4**: 32 µg/mL Fe-Ni ANPs; **5**: 128 µg/mL Fe-Ni ANPs; **6**: positive control. GC: growth control; PC: positive control (3 µM H_2_O_2_). Data represent the average value of three independent XTT and clonogenic assays. Error bars: the standard deviation of the mean. Asterisk: significantly different from the control (*: *p* < 0.05; **: *p* < 0.01;***: *p* < 0.001).

The XTT assay results (Figure 2A) showed that the first four concentrations of Fe-Ni ANPs did not cause a statistically significant change in cell viability versus the growth control group. The cell viability decreased significantly starting from 4 µg/mL Fe-Ni ANPs (viability equal to 76.899 ± 6.633 (*p* < 0.05) compared to control) and above. The cell viability was determined as 24.069 ± 2.416 (*p* < 0.001) in the 128 µg/mL Fe-Ni ANPs concentration group. The mean IC50 value was calculated from three different XTT repeat results as 37.409 ± 2.032 µg/mL (r^2^ = 0.987 ± 0.021).

The clonogenic assay results (Figure 2B,C) show that the first four concentrations of Fe-Ni ANPs did not cause a statistically significant change in cell viability versus the control group, similar to the XTT assay. The cell viability decreased significantly starting from 4 µg/mL Fe-Ni ANPs (viability equal to 68.667 ± 3.055 (*p* < 0.01) compared to control) and above. The cell viability was 22.000 ± 1.732 (*p* < 0.001) at 128 µg/mL Fe-Ni ANPs. The mean IC50 value was calculated from three different clonogenic assay repeat results as 38.687 ± 1.739 µg/mL (r^2^ = 0.996 ± 0.003).

The IC50 value of 38.048 µg/mL was used in other experiments and was determined by the average IC50 values calculated from XTT and clonogenic methods. The concentrations used in other experiments in this study are shown in Table 1.

### 3.3. Genotoxicity

**Figure 3:**
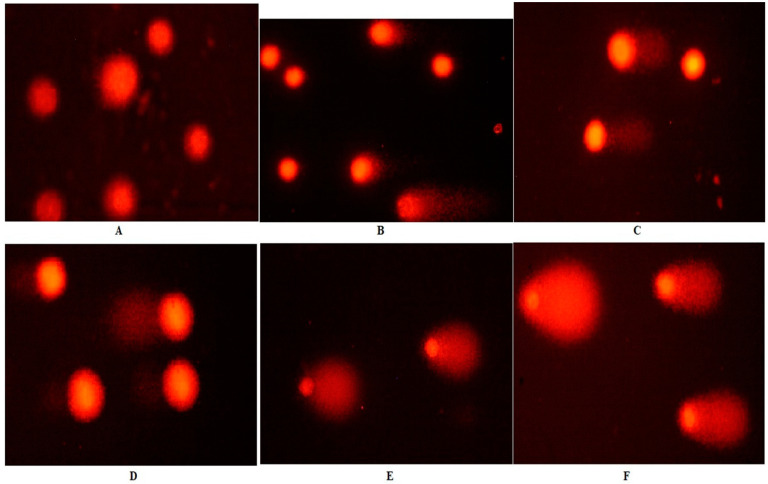
Sample images from the comet assay. (**A**) Growth control; (**B**) IC12.5; (**C**) I C25; (**D**) IC50; (**E**) IC75; (**F**) positive control (200 µM H_2_O_2_).

The comet assay evaluated the DNA damage effects of Fe-Ni ANPs with tail length, tail DNA %, and Olive tail moment (OTM) parameters. The tail length value was determined as 9.531 ± 2.632 µm in the control group and increased significantly (*p* < 0.01) to 67.846 ± 3.976 µm at the IC75 concentration of Fe-Ni ANPs. Similarly, all Fe-Ni ANPs concentrations used in all three comet parameters caused a significant (*p* < 0.001) increase (Table 2 and Figure 3).

**Figure 4:**
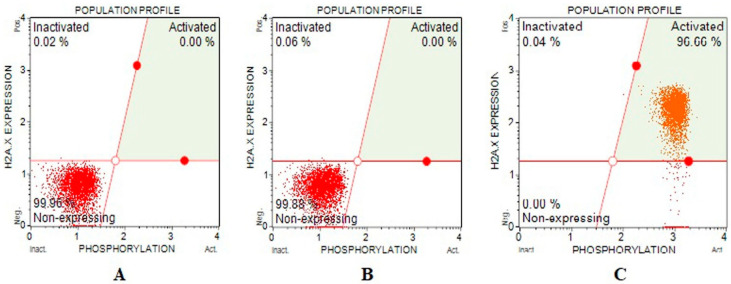
Sample plots to γH2AX assay from Muse Cell Analyzer. (**A**) Growth control; (**B**) IC25; (**C**) IC75.

DNA double-strand breaks effects of Fe-Ni ANPs were evaluated with a γH2AX kit. The amount of activated γH2AX determined is proportional to the DNA double-strand breaks (DSB). The percentage of cells containing activated γH2AX at IC75 concentration groups was determined as 96.493 ± 1.198, and the same parameter value was determined as 0 in both growth control and IC25 concentration groups. Similarly, the non-expressing γH2AX level was determined as 99.82 ± 0.192 in the growth control group, 99.853 ± 0.011 in the IC25 concentration group, and 0 in the IC 75 concentration group (Table 3 and Figure 4). The amount of DNA DBS increased significantly in the IC75 concentration group.

### 3.4. Cell Death Mode

**Figure 5:**
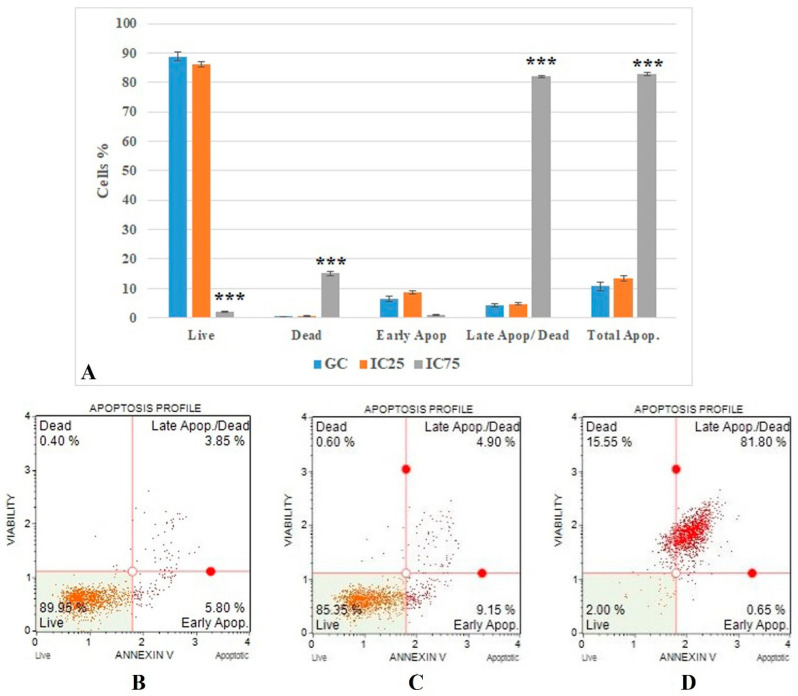
(**A**) Results of cell death mode from Annexin V analysis. GC: growth control; Apop: apoptotic. Data represent the average value of three independent Annexin V analyses. Error bars: the standard deviation of the mean. Asterisk: significantly different from the control (***: *p* < 0.001). (**B**–**D**): Sample plots to Annexin V analysis from Muse Cell Analyzer. (**B**) Growth control; (**C**) IC25; (**D**) IC75.

The flow cytometric Annexin V results (Figure 5) show that the IC75 concentration of Fe-Ni ANPs significantly increased as a percentage of late apoptotic/dead cells compared to the growth control group (*p* < 0.001). The percentage of cells in this parameter was determined as 4.225 ± 0.53 in the growth control group. This value was 82.075 ± 0.388 in the IC75 concentration Fe-Ni ANP group.

**Figure 6:**
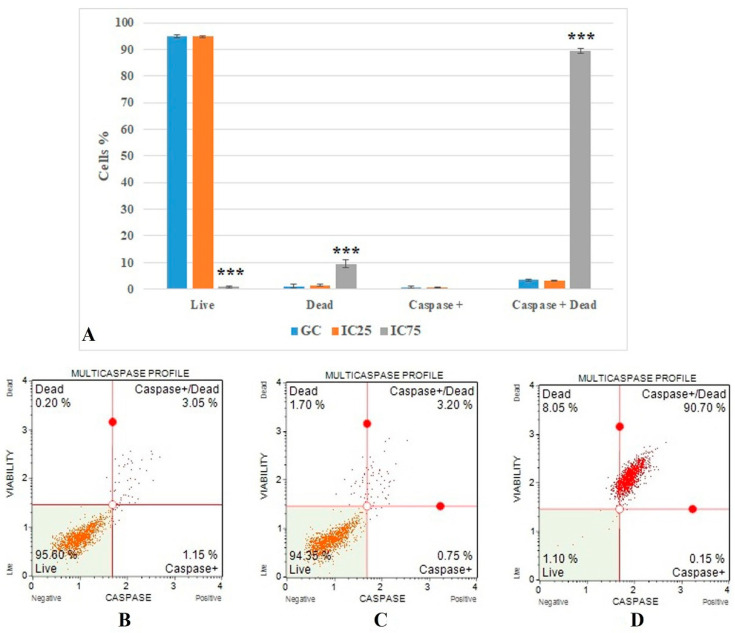
(**A**) Results of cell death mode from multicaspase analysis. GC: growth control. Data represent the average value of three independent multicaspase analyses. Error bars: the standard deviation of the mean. Asterisk: significantly different from the control (***: *p* < 0.001). (**B**–**D**): Sample plots to multicaspase analysis from Muse Cell Analyzer. (**B**) Growth control; (**C**) IC25; (**D**) IC75.

The flow cytometric multicaspase results (Figure 6) show the caspase + dead cells value. It was 3.366 ± 0.284 in the growth control group and increased significantly (*p* < 0.001) in the Fe-Ni ANPs IC75 concentration (89.483 ± 1.061).

### 3.5. Cell Cycle Arrested Analysis

**Figure 7:**
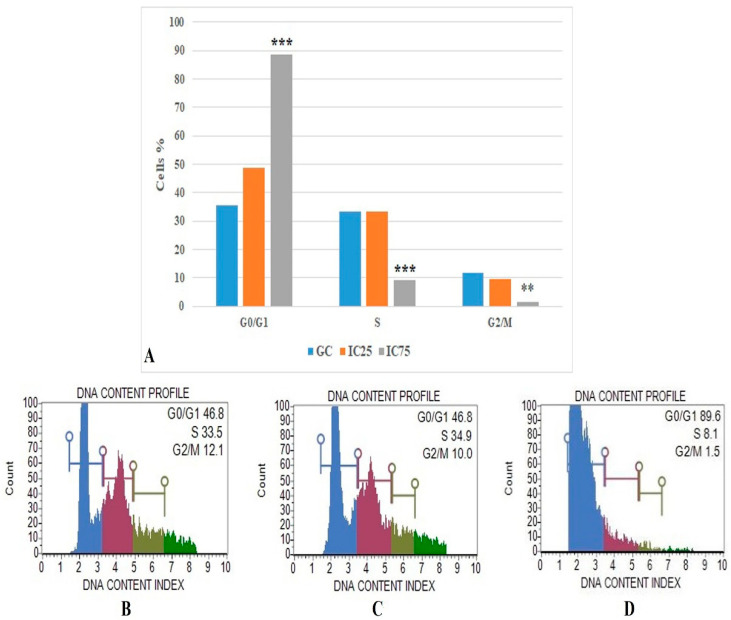
(**A**) Results of cell cycle arrested analysis. GC: growth control. Data represent the average value of three independent cell cycle arrested analyses. Error bars: the standard deviation of the mean. Asterisk: significantly different from the control (***: *p* < 0.001). (**B**–**D**): Sample plots to cell cycle arrested analysis from Muse Cell Analyzer. (**B**) Growth control; (**C**) IC25; (**D**) IC75.

The flow cytometric cell cycle analysis results were based on DNA content profile (Figure 7) and show that the IC75 concentration of Fe-Ni ANPs maintains 88.633 ± 1.001 percent of cells at the G0/G1 stage. The same value was 46.933 ± 0.321 for the growth control group. Versus the growth control group, the IC75 concentration of Fe-Ni ANPs increased the G0/G1 cell ratio in a statistically significant way (*p* < 0.001).

### 3.6. Amount of Intracellular ROS

**Figure 8:**
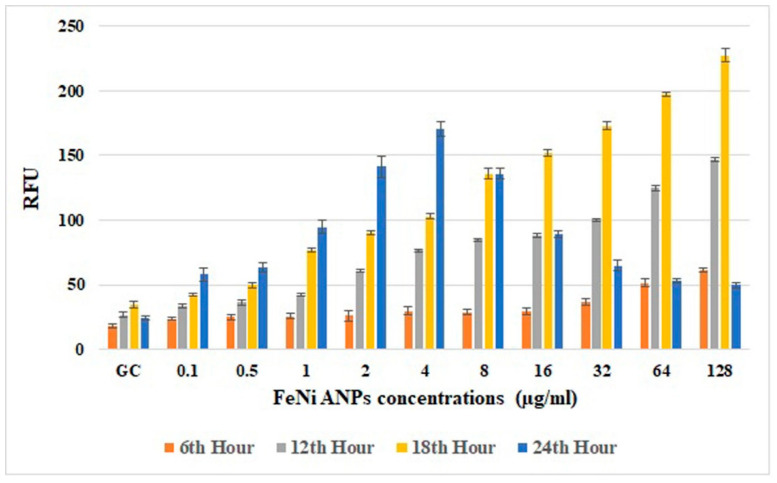
Results of intracellular ROS assay. RFU: relative fluorescence unit.

The amounts of intracellular ROS (Figure 8) analyzed using RFU values were generally increased by Fe-Ni ANPs. An increased RFU value reflects an increased amount of intracellular ROS. Versus the growth control group at hour 18, the RFU value increased in a statistically significant way with Fe-Ni ANPs at 1 µg/mL and above (*p* < 0.001).

## 4. Discussion

Nanomaterials’ biological and toxicological effects should be evaluated for rapidly developing nanotechnological products to work safely and as intended. Here, the toxic effects of iron-nickel nanoparticles have different uses and were assessed on Beas-2B cells in vitro. Monoculture studies lack explanations for how nanoparticles might interact with a particular organ of the body [37,48]. However, monoculture systems can provide high efficiency for nanotoxicology research. There are many studies evaluating the genotoxic and cytotoxic activities of different nanoparticles using the Beas-2B cells used here [49,50,51,52,53,54,55,56]. Beas-2B cells (human healthy bronchial epithelial cells) were used to model the lungs, an essential gateway for nanoparticle exposure. There are many use areas for Fe-Ni ANPs. The air contains many nanoparticles, such as Fe and Ni, and can be a route of exposure. Haghani et al. [57] and Maher et al. [58] showed that we could be exposed to nanoparticles, such as Fe and Ni, in the air. That is our rationale for using Beas 2B cells.

The physicochemical properties of nanoparticles affect their behavior in biological environments [59]. Fe-Ni ANPs’ zeta potential values were determined to be −24.8 mV in distilled water and −20.2 mV in RPMI-1640 medium. As stated by Bhattacharjee [59], changes in pH and ionic strength caused different zeta potential values between solutions. Our zeta potential value shows that Fe-Ni ANPs are a moderately stable colloid [59,60]. In their study, Goodman et al. [61] stated that negative zeta potential valuable gold nanoparticles are not toxic. Similarly, Shao et al. [62] stated that nanoparticles with positive surface charges are more toxic. Similar results to our study were shown by Zhang et al. [63], where anionic iron oxide nanoparticles might also have potentially toxic effects via their intracellular entry mechanisms. However, Shao et al. [62] showed that negatively charged nanoparticles also have toxic activity, although less than positively charged nanoparticles. In addition, the toxic activities of negatively charged nanoparticles have been shown in our previous studies [31]. Many articles have reported the toxic effects of negatively charged nanoparticles as in our study [64,65,66,67]. Although the Fe-Ni ANPs used in our study are negatively charged, they can have toxic effects. Our XRD results were almost identical to those of Chau [68] with Fe-Ni nanoparticles. The XRD results confirm that our material is in the form of Fe-Ni bimetallic nanoparticles (with lattice parameter a = 3.59 Å). In addition, our intracellular TEM images show that Fe-Ni ANPs are concentrated in the cytoplasm.

Particle size is also one of the critical parameters of nanotoxicity. The present study measured particle size at approximately 180 nm by DLS and 42 nm by TEM. As highlighted by Bhattacharjee [59], “such information from TEM images often do not corroborate well with data obtained from DLS as the latter is an intensity-based technique whereas TEM is a number-based one making them fundamentally different.” DLS data are more reliable because they are based on a much larger number of measurements in terms of particle size [59]. However, while hydrodynamic particle size is measured in DLS analysis, TEM results are obtained from the material under vacuum. Therefore, finding differences between the results of the two techniques seems normal. The DLS results’ slightly larger particle size is likely due to unpredictable agglomeration [59,69]. Shalini et al. [70] stated that 187–1039 nm ZnO nanoparticles showed cytotoxic effects in human peripheral blood lymphocytes. It is understood that the Fe-Ni ANPs size used in the present study does not prevent their toxic activities.

Colorimetric methods are frequently used to determine the in vitro cytotoxic activity of nanoparticles [37]. We used XTT to measure the cytotoxicity activity of Fe-Ni ANPs. The nanoparticle background absorbance values, which may cause false positives, were considered as stated by Stone et al. [37]. The clonogenic method, also known as colony-forming efficacy (CFE), was also used to increase the reliability of cytotoxicity results [71]. The compatibility of the IC50 values obtained from the two methods with each other increases the reliability of our cytotoxicity results. This study determined the mean IC50 value obtained from both methods to be 38,048 µg/mL. During the literature review, no similar study was found with Fe-Ni ANPs, but there are studies performed with different nanoparticles. Ahamed [72] evaluated the cytotoxicity of nickel nanoparticles with the MTT method in his study in A549 cells. After 24 h of exposure, the cell viability rate was 21% at 25 µg/mL. Capasso et al. [34] used AB (Alamar blue) to show that nickel oxide nanoparticles at 100 µg/mL reduced the viability of Beas-2B cells by only 30% after 24 h of exposure. In different studies, the cytotoxic activities of nanoparticles, such as nickel and nickel oxide, were demonstrated using different cell lines. [73].

Rajiv et al. [74] stated that a 100 µg/mL concentration of iron (III) oxide decreased cell viability by approximately 50% after 24 h of exposure in human lymphocyte cells. Dönmez Gungunes et al. [75] stated that iron oxide nanoparticles at a concentration of 100 µg/mL for 24 h caused a decrease in cell viability of approximately 30% in mouse dermal fibroblast cells and a decrease of roughly 50% in human periodontal ligament fibroblasts cells. In different studies, cytotoxic activities of nanoparticles, such as iron, iron oxide, and derivatives, have been demonstrated using different cell lines [76].

Concentrations of cytotoxic activity for different nanoparticles containing either iron or nickel differ in studies. We think this difference is due to the different cell lines and methods used. However, the most important reason why the IC50 value we obtained in our study is not exactly the same as in other studies is that there is no further study using the Fe-Ni ANPs that we used in our study. However, with the IC50 value of 38.048 µg/mL, which we determined in our study, we can state that Fe-Ni ANPs have cytotoxic activity in Beas-2B cells after 24 h of exposure as in other related nanoparticles.

DNA strand breaks can easily occur after exposure to genotoxic components. Methods such as the comet test determine DNA damage through DNA strand breaks and are frequently used to investigate nanoparticles’ genotoxic activities. In this study, an alkaline comet assay was used to determine single strand breaks (SSB). Although there is no genotoxicity study with Fe-Ni ANPs, there are many studies investigating the genotoxic activities of related nanoparticles by the comet method [77]. Mesárošová et al. [78] investigated the genotoxic activities of iron oxide nanoparticles coated with different materials in their study using A549 cells by the comet method and determined genotoxic activity at a concentration of approximately 23 µg/mL. Radeloff et al. [79] showed that iron oxide nanoparticles had no genotoxic activity even after exposure to a concentration of 1.5 mM in human adipose-tissue-derived stromal cells. Similarly, studies have shown the genotoxic activity of different nickel-containing nanoparticles [80,81,82].

Here, Fe-Ni ANPs showed genotoxic effects starting from our lowest concentration of 9.512 µg/mL (IC12.5), similar to other studies performed with different nanoparticles. Determining the amount of activated γH2AX is another method frequently used to determine the genotoxic activity of many different agents, including nanoparticles [83,84]. In our study, the DNA SSBs parameter, a marker of genotoxicity, was determined by the alkaline comet assay, while another important marker of genotoxicity, DSBs, was determined using the γH2AX parameter. The increased amount of activated γH2AX indicates that high concentrations of Fe-Ni ANPs cause DNA DSBs, especially in the IC 75 concentration group. The general tendency is to prefer concentrations above IC50 so that the results can be presented more clearly, especially in flow cytometric studies [85,86,87]. For this reason, concentrations below and above the IC50 concentrations were preferred, especially when evaluating our study’s flow cytometric methods results.

Different methods determined the cytotoxic and genotoxic activities of Fe-Ni ANPs in Beas-2B cells. The basic mechanism underlying the toxic activities of different nanoparticles is oxidative stress and apoptosis [88]. Here, cell death mode was determined by Annexin V analysis to investigate the apoptotic activity of Fe-Ni ANPs. The results show that Fe-Ni ANPs at IC75 (57.072 µg/mL) induced apoptosis in Beas-2B cells. Our apoptosis findings were supported by multicaspase analysis and it was determined that Fe-Ni ANPs showed an apoptotic effect through the caspase-dependent apoptotic pathway. In addition, the results obtained with the cell cycle arrested method show that the cell cycle is arrested in the G0/G1 phase by Fe-Ni ANPs. Cell cycle arrest in the G0/G1 phase is another factor that triggers apoptosis [71]. Similar results to our findings on apoptosis were also obtained in studies using different Fe- or Ni-containing nanoparticles [88,89,90,91,92,93,94]. DNA damage starts from the IC 12.5 concentration, but DSBs, which can represent more advanced DNA damages, begin to be seen at the IC75 concentration via γH2AX. However, our findings obtained with annexin V, multicaspase, and cell cycle arrested methods also show that Fe-Ni ANPs apoptosis induction is at the IC75 concentration. This suggests that cells are driven into apoptosis, especially due to DSBs occurring in DNA.

Apoptosis is a cell death pathway that usually occurs via activation of certain intracellular signals against unrepaired DNA damage in cells. ROS generation in the cell may be one of the main factors initiating apoptosis [92]. Our ROS results show a significant increase in intracellular ROS, especially at the 18th hour of exposure to Fe-Ni ANPs. In many studies, the amount of intracellular ROS determined by similar methods is based on the results in a certain time period. In our study, intracellular ROS amounts reached their highest levels in the measurements at the 18th hour. In our measurements at the 24th hour, we think that the decrease in RFU values is due to the decrease in the number of viable cells. Decreased cell viability also causes a decrease in intracellular ROS production. In addition, the decrease in the number of viable cells also means that the cellular esterases that will enable the formation of DCF will decrease, which is used as a ROS marker in the method (Figure 8). Studies with iron-containing nanoparticles have shown an increase in the amount of intracellular ROS through Fenton reactions [92]. Moreover, Wu et al. [95] stated that iron nanoparticles can cause H_2_O_2_ degradation with peroxidase-like activity after being trapped in acidic lysosomes. The same study emphasized that, although acidic lysosomes are not trapped, they can increase the amount of intracellular ROS by acting like catalase in the neutral cytosol. In general, nanoparticles mediate oxidative stress by increasing lipid peroxidation and MDA levels while decreasing cellular antioxidant levels [74]. In addition, there are studies showing that nickel-containing nanoparticles also mediate oxidative stress by reducing antioxidant levels [72,73,96]. Our study’s increased intracellular ROS findings show that Fe-Ni ANPs cause oxidative stress through the above-mentioned mechanisms. Increasing genotoxic damage due to the increased amount of ROS led the cells to apoptosis, thus causing the Fe-Ni ANPs to show cytotoxic, genotoxic, and apoptotic effects.

In conclusion, inhalation plays a critical role in human exposure to nanoparticles, and Fe-Ni ANPs have cytotoxic and genotoxic activity in Beas-2B cells-healthy human lung bronchial epithelial cells. The XTT and clonogenic methods analyzed the cytotoxic activity, and the IC 50 value was 38.048 µg/mL for Beas-2B cells after 24 h of exposure to Fe-Ni ANPs. Comet assay and flowcytometric γH2AX methods were used for the genotoxic activity. The comet parameters began to increase from the IC12.5 value of the selected concentration. In addition, Fe-Ni ANPs have been shown to have genotoxic activity by determining the increase in flow cytometric γH2AX parameters at the IC75 concentration. As the basic mechanism of genotoxic and cytotoxic activity, it has been shown that the amount of intracellular ROS is increased by Fe-Ni ANPs. As a mechanism of cytotoxicity, it was demonstrated by Annexin V and multicaspase assay that Fe-Ni ANPs induced apoptosis. In addition, it was determined that Fe-Ni ANPs stopped the cells at the G0/G1 step with the cell cycle arrest method, which supports the induction of apoptosis. We believe that the results expand knowledge regarding the toxicity of bimetallic Fe-Ni ANPs and their possible mechanisms.

## Figures and Tables

**Table 1:** Concentrations used in the experiments and their definitions.

The Definition Used	Concentrations µg/mL	The Definition Used	Concentrations µg/mL
**IC12.5**	9.512	**IC50**	38.048
**IC25**	19.024	**IC75**	57.072

**Table 2:** Results of genotoxicity from comet assay.

Con.	Tail Length (µm)			Tail DNA %			OTM		
	Mean ± SD	Min.	Max.	Mean ± SD	Min.	Max.	Mean ± SD	Min.	Max.
**GC**	9.531 ± 2.632	3.987	21.964	9.953 ± 1.866	4.314	14.150	2.050 ± 0.725	0.372	4.004
**IC12.5**	30.016 ± 6.270 ***	15.967	54.021	31.704 ± 8.395 ***	11.327	58.414	10.591 ± 2.885 ***	2.380	20.445
**IC25**	45.200 ± 6.879 ***	32.678	80.324	44.183 ± 7.224 ***	32.360	77.535	17.114 ± 3.788 ***	10.387	34.115
**IC50**	59.842 ± 4.032 ***	46.657	66.546	62.833 ± 6.067 ***	50.354	80.535	21.720 ± 3.451 ***	15.106	32.214
**IC75**	67.846 ± 3.976 ***	55.967	73.657	63.833 ± 6.058 ***	51.354	81.535	24.619 ± 3.645 ***	17.460	35.875
**PC**	71.958 ± 6.137	55.478	95.331	73.519 ± 8.196	56.012	91.920	25.484 ± 4.060	15.594	47.137

Con: Fe-Ni ANPs concentration; GC: growth control; PC: positive control (200 µM H_2_O_2_); OTM: Olive tail moment. Asterisk: significantly different from the GC (***: *p* < 0.001).

**Table 3:** Results of genotoxicity from γH2AX assay.

Cells %			
	Activated γH2AX	Inactivated γH2AX	Non-Expressing γH2AX
**GC**	0	0.006 ± 0.015	99.82 ± 0.192
**IC25**	0	0.046 ± 0.015	99.853 ± 0.011
**IC75**	96.493 ± 1.198	0.82 ± 1.368	0

GC: growth control.

## Data Availability

Not applicable.
